# The effect of adding Bi^3+^ on the performance of a newly developed iron–copper redox flow battery†

**DOI:** 10.1039/c7ra12926b

**Published:** 2018-02-23

**Authors:** Daniel Manaye Kabtamu, Guan-Yi Lin, Yu-Chung Chang, Hsueh-Yu Chen, Hsin-Chih Huang, Ning-Yih Hsu, Yi-Sin Chou, Hwa-Jou Wei, Chen-Hao Wang

**Affiliations:** Department of Materials Science and Engineering, National Taiwan University of Science and Technology 10607 Taipei Taiwan chwang@mail.ntust.edu.tw +886-2-2737-6544 +886-2-2730-3715; Institute of Nuclear Energy Research, Atomic Energy Council, Executive Yuan 32546 Taoyuan Taiwan

## Abstract

In this paper, we propose a new, abundant, cost-effective, non-toxic, and environmentally benign iron–copper redox flow battery (Fe/Cu RFB), which employs Fe^2+^/Fe^3+^ and Cu^+^/Cu^0^ as the positive and negative electrolytes, respectively. The effect of graphite felt (GF) electrode modification and addition of Bi^3+^ into the electrolytes on the performance of the Fe/Cu RFB were investigated. It was found that the cell containing Bi^3+^ in the electrolytes revealed higher coulombic efficiency (89.18%) and energy efficiency (35.24%) than the cell without Bi^3+^ (CE = 84.10% and EE = 34.43%) at 20 mA cm^−2^. This is because after adding Bi^3+^, Cu metal precipitation was not observed on the electrode surface, which indicates that the deposition process was potentially reversible on the electrode material, thus leading to enhanced performance of the battery. Furthermore, the efficiencies of the battery are stable over 10 cycles, which demonstrates that Fe/Cu RFB exhibits good stability on the microwave heat treated GF plus one layer microwave heat treated carbon paper (HT-GF + HT-CP) electrode after adding Bi^3+^ into the electrolytes.

## Abstract 2

In this paper, we propose a new, abundant, cost-effective, non-toxic, and environmentally benign iron–copper redox flow battery (Fe/Cu RFB), which employs Fe^2+^/Fe^3+^ and Cu^+^/Cu^0^ as the positive and negative electrolytes, respectively.

## Graphical abstract

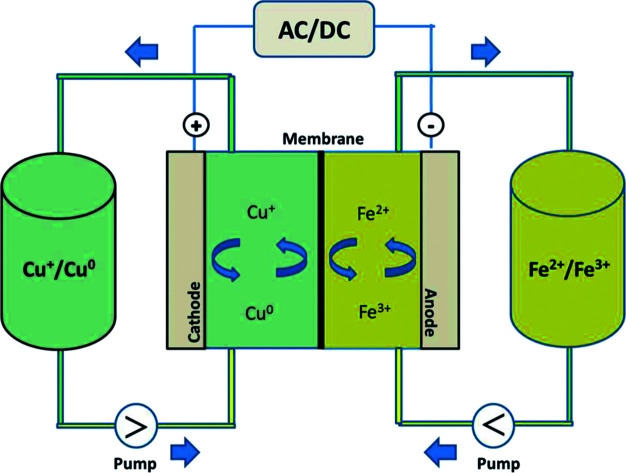

## 1. Introduction

Due to the environmental concerns over burning of fossil fuels and their limited resources, along with the rising energy demands for the increasing global population, the utilization of clean and renewable energy generated from renewable sources, such as wind and solar, have attracted considerable social and scientific attention in recent years. However, the varied nature of such intermittent sustainable energy sources makes it difficult to integrate this valuable energy into the electrical grid supply. Therefore, a large-scale electrical energy storage system is needed to alleviate this problem.^1–4^

Redox flow batteries (RFBs) are one of the most promising energy storage system technologies owing to their attractive features such as high efficiency, intrinsic safety, short response time, deep discharge ability, long cycle life, and tolerance to over-charge and over-discharge.^2,3,5,6^ In redox flow batteries, large amounts of energy are stored in a pair of reduced and oxidized electro-active species dissolved in two separate liquid electrolytes alleged outside of the cell-stack. This permits us to independently scale up the power and energy components of the system (up to multi-MW and multi-MW h, respectively) by maintaining all of the electro-active species in a fluid form.^7–10^ The decoupling of the energy storage and power generation supplies provides a substantial design opportunity to independently scale-up the power capability or energy capacity for various energy storage system applications.^11^

Among the numerous types of RFBs that have been reported to date,^12,13^ all-vanadium redox flow batteries (VRFBs), originally proposed by Skyllas-Kazacos *et al.*,^14^ are highly suitable and widely studied for large-scale energy storage systems because of the good electrochemical reversibility of vanadium redox couples. In addition, they use active species of the same vanadium metal as the electrolytes, which substantially minimizes the problem of active component crossover contamination across the membrane.^15,16^ However, the limited earth-abundance and high cost of vanadium and the relatively low energy efficiency of the VRFBs still hinder their widespread commercial adoption.^6^ A feasible strategy to reduce the cost of RFBs should therefore be considered in terms of materials, cell configurations, and fabrication processes. Therefore, developing alternative low-cost and low-toxicity electrolyte materials is critical in RFBs.^1^

The all-iron redox flow battery (Fe RFB) was first pioneered by Hruska and Savinell in 1981.^17^ It utilizes the Fe^3+^/Fe^2+^ and Fe^2+^/Fe redox couples in positive and negative electrolytes, respectively, and is suitable for grid-scale energy storage applications. This is because of the availability, low-cost, and environmentally benign status of the active element (Fe).^18^ However, there are challenges in achieving the successful commercialization of this redox flow battery, such as low coulombic efficiency, hydrogen evolution and poor deposition/dissolution kinetics at the negative electrode, high self-discharge rate, and poor cycle life.^19^

The iron–chromium redox flow battery (Fe/Cr RFB) is an attractive alternative to the traditional RFBs. The Fe/Cr RFB has been considered as the first true RFB and utilizes low-cost and completely soluble redox-active materials in both the positive and negative electrolytes.^13^ In the Fe/Cr RFB battery, the Fe^2+^/Fe^3+^ redox couple is used in the positive half-cell while the Cr^2+^/Cr^3+^ redox couple is used in the negative half-cell. The Fe^2+^/Fe^3+^ redox couple exhibits a high electrochemical reversibility on carbon and graphite felt electrodes at the positive side. However, a catalyst is required at the negative side due to the relatively slow kinetics of the Cr^2+^/Cr^3+^ couple on these electrodes. Moreover, hydrogen evolution occurs as a side reaction to the reduction of the Cr^3+^ species, which reduces the coulombic efficiency and causes capacity decay.^20^

Recently, all-copper redox flow batteries (Cu RFB) have attracted tremendous attention for practical applications because copper is highly abundant with high purity and also, it is inexpensive, less toxic, and highly soluble in water.^21^ Furthermore, the carbon electrodes and the polymeric separators that can be applied in the all-copper system are also relatively cheap materials and the heat exchangers are eliminated.^21^ RFBs based on the chemistry of copper employ the three oxidation states of copper, namely, Cu(s), Cu^+^ and Cu^2+^. Among these, the Cu^+^ ion is unstable and undergoes disproportionation to form Cu(s) and Cu^2+^ at ambient temperature and pressure. However, Cu^+^ can be stabilized by forming a complex with suitable complexing agents such as chloride ions. Previous studies have shown that Cu^+^ the complexes CuCl(aq) and/or CuCl_2_^−^ are dominant in dilute acid chloride solutions, while high order chloro-complexes, for instance, CuCl_3_^2−^ and/or CuCl_4_^3−^ become more stable at higher concentrations of chloride ions.^22^ One of the main limitations of an all-copper redox flow battery is a relatively low cell voltage (0.6 V), which is considerably lower than those of other traditional RFBs (>1.15 V).^23^

**Fig. 1:**
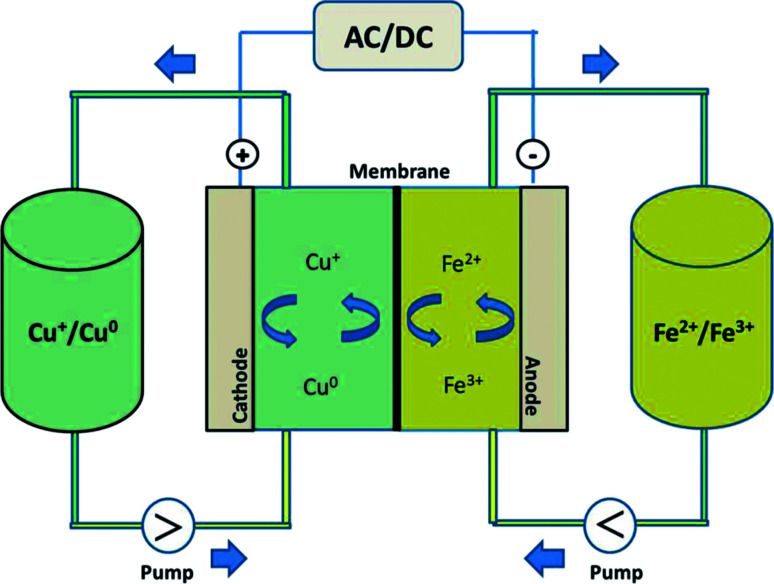
Schematic illustration of the experimental set-up of a Fe/Cu redox flow battery.

In this study, by combining the numerous benefits of the iron and copper redox flow battery systems, we propose a new, abundant, cost-effective, non-toxic, and environmentally benign iron–copper redox flow battery (Fe/Cu RFB), which employs Fe^2+^/Fe^3+^ and Cu^+^/Cu^0^ as the positive and negative electrolytes, respectively, as shown schematically in Fig. 1. A standard cell voltage of 0.25 V was produced through the following electrochemical reactions in a Fe/Cu RFB:

Positive electrode:

1 Fe^2+^ ↔ Fe^3+^ + e^−^, *E*^0^ = 0.77 V

Negative electrode:

2 Cu^+^ + e^−^ ↔ Cu^0^, *E*^0^ = 0.52 V

Cell reaction:

3 Fe^2+^ + Cu^+^ ↔ Fe^3+^ + Cu^0^, *E*^0^ = 0.25 V *vs.* SHE

During the charging process, ferrous ions (Fe^2+^) are oxidized to ferric ions (Fe^3+^) in the positive half-cell, while cupreous ions (Cu^+^) are reduced to copper metal (Cu^0^) in the negative half-cell. When the battery is in the discharge process, reversed reactions take place. The effect of GF electrode modification and addition of Bi^3+^ to the electrolytes on the performance of the Fe/Cu RFB are investigated. It was found that the cell containing Bi^3+^ in the electrolytes revealed higher coulombic efficiency (CE = 89.18%) and energy efficiency (EE = 35.24%) than the cell without Bi^3+^ (CE = 84.10% and EE = 34.43%) at a current density of 20 mA cm^−2^. Furthermore, after adding Bi^3+^ into the electrolytes, the efficiencies of the battery are stable over 10 cycles, which demonstrates that Fe/Cu RFB has a good stability on the microwave heat treated graphite felt plus one layer microwave heat treated carbon paper (HT-GF + HT-CP) electrode under these conditions.

The main shortcoming of the Fe/Cu RFB is its relatively low cell voltage. The cell voltage is significantly lower than those of other RFB systems, leading in principle, to lower values of energy density. However, high solubility of iron and copper in aqueous solution (by increasing the concentration of active species used in the electrolyte) may overcome this issue in the future. The use of high amounts of iron and copper are not a key issue in terms of cost since these materials are abundant, less toxic, and cheaper than other redox pairs commonly used in these technologies. For example, compared with vanadium metal, the use of iron and copper are more attractive for broad market penetration as they have a lower cost ($2.4 kg^−1^ of iron and $7.4 kg^−1^ of copper *versus* $23.5 kg^−1^ of vanadium).^20,23^ The major conclusion drawn from our study is that a small cell voltage does not render the Fe/Cu RFBs ineffective to store energy efficiently. In addition, the low cell voltage clearly benefits the coulombic efficiency of the system with slight tendency for electrode side reactions to occur at a higher current densities.^24^

## 2. Experimental

### 2.1. Preparation of electrolytes

In this study, all reagents were used without further treatment. The iron electrolyte was prepared by dissolving 1.5 M of FeCl_2_ in 3 M HCl solution, while the copper electrolyte was prepared by dissolving 1.8 M of copper(i) chloride (CuCl) in mixed supporting electrolyte (CaCl_2_ + HCl). In order to increase the concentration of chloride in copper electrolyte solution, 2.4 M of CaCl_2_ and 2.4 M of HCl were utilized as a mixed supporting electrolyte. All solutions were prepared with distilled water at room temperature.

### 2.2. Electrochemical measurements

Cyclic voltammetry (CV) measurements were performed using a typical three-electrode cell at room temperature. A platinum wire and Ag/AgCl were used as the counter and reference electrodes, respectively. The working electrode was a glassy carbon (GC) disk with a geometric surface area of 0.196 cm^2^. For the iron half-cell, a CV test was performed in 1.5 M FeCl_2_ in a 3 M HCl solution in the potential range between −0.5 and 1.2 V *versus* Ag/AgCl, while for copper half-cell, a CV test was performed in 1.8 M CuCl in a mixed supporting electrolyte (2.4 M CaCl_2_ + 2.4 M HCl) in the potential range between −0.7 to 1.0 V *versus* Ag/AgCl. During the tests, various scan rates (10–70 mV s^−1^) were used. A CV test was also performed for both iron and copper half-cell electrolytes without and with 0.01 M Bi_2_O_3_ at a scan rate of 50 mV s^−1^. The electrolytes were nitrogen-purged to avoid undesired oxidation. All electrochemical measurements were performed using a potential station (Solartron 1280C).

### 2.3. Charge–discharge tests of Fe/Cu RFB

A Fe/Cu RFB single cell test was performed using a 25 cm^2^ (5 cm × 5 cm) area of the modified graphite felt (GF) electrodes for both the negative and positive sides. A modified GF electrode was prepared using microwave thermal treatment. A pristine GF and one layer carbon paper (CP) were heat treated at 400 °C for 15 min and then cooled to ambient temperature before use. The main objective of the heat treatment of pristine GF and CP was to improve their electrochemical activity and wettability. A pristine Nafion 117 membrane was used as a separator. The cell was connected to two glass reservoirs holding a balanced volume of electrolytes (50 mL) on both negative and positive sides. The concentration of electrolytes was 1.5 M FeCl_2_ in 3 M HCl solution and 1.8 M CuCl in a mixed supporting electrolyte (2.4 M CaCl_2_ + 2.4 M HCl) solution in the positive and negative sides, respectively. Then, 0.01 M Bi_2_O_3_ was added in both the anolyte and catholyte. The electrolyte flow rate for each half-cell was 30 mL min^−1^. The charge–discharge profiles of the Fe/Cu RFB were evaluated though a potential station (Solartron 1280C) at a current density of 20 mA cm^−2^ within a fixed potential range of 0–1.2 V.

## 3. Results and discussion

**Fig. 2:**
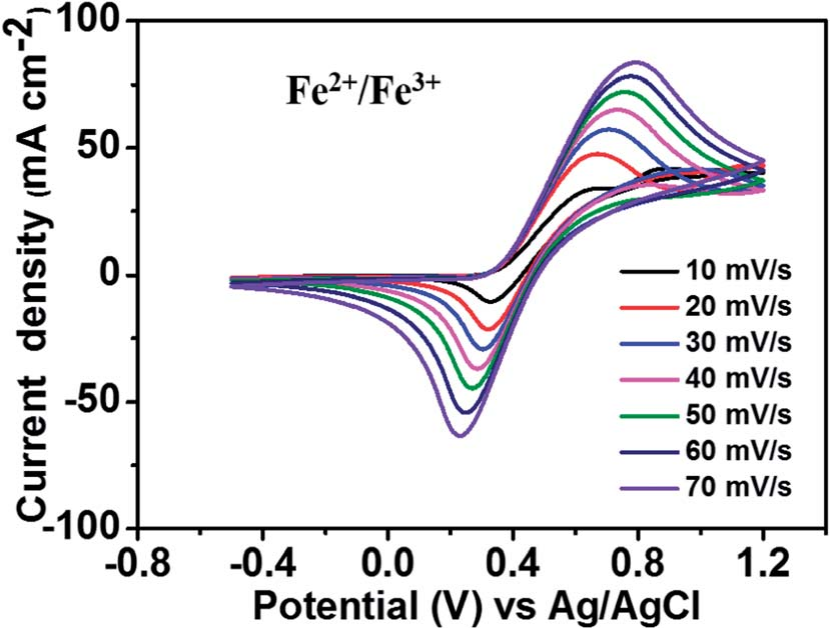
CV curves of 1.5 M FeCl_2_ in a 3 M HCl using GC electrode at various scan rates.

Fig. 2 depicts the CV curves of the Fe^2+^/Fe^3+^ redox couple using a GC electrode with various scan rates between −0.5 and 1.2 V (*vs.* Ag/AgCl) at room temperature. The current density was normalized to the geometrical area of the working electrode. As shown in the figure, increasing the scan rate from 10 to 70 mV s^−1^ results in an increase in the anodic and cathodic peak current densities. Moreover, the anodic peak potential that appears at about 0.68–0.78 V corresponds to the oxidation of Fe^2+^ to Fe^3+^. The corresponding cathodic peak potential occurs at about 0.23–0.33 V during the reverse scan. The redox peak potential separation related to the redox couple Fe^2+^/Fe^3+^ is around 0.4 V, indicating that the Fe^2+^/Fe^3+^ redox couple has sufficient reversibility to be used as the positive electrolyte for the proposed redox flow battery system.

**Fig. 3:**
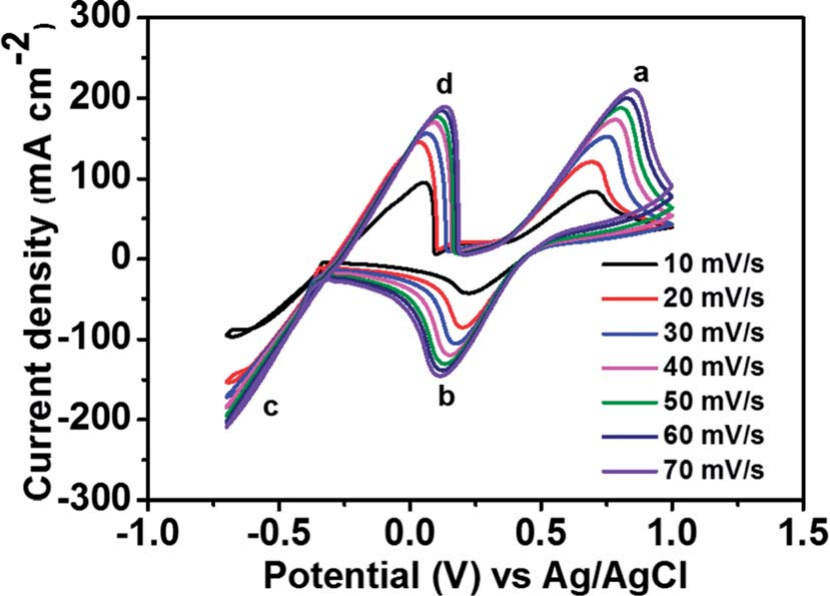
CV curves of 1.8 M CuCl in a mixed supporting electrolyte (2.4 M CaCl_2_ + 2.4 M HCl) solution using GC electrode at various scan rates.

To determine the potential region of the redox reactions, the CV curves of 1.8 M CuCl in 2.4 M HCl/2.4 M CaCl_2_ electrolyte solution were performed using a GC working electrode at different scan rates, as shown in Fig. 3, in which the potential window for Cu^+^/Cu^0^ and Cu^2+^/Cu^+^ was over 1.6 V. As observed, peak (a) displays the oxidation of Cu^+^ to Cu^2+^ and peak (b) shows the reduction of Cu^2+^ to Cu^+^. Peak (c) suggests the solid Cu formation on the GC electrode surface on the negative side. The sharp decrease in current indicates the typical nucleation loop related to the Cu metal electrodeposition onto the GC electrode. Once nucleation occurs, no difference is apparent between the various scan rates used. The final peak (d), suggests the dissolution of Cu^0^ that is electrodeposited onto the GC electrode, which oxidizes back to Cu^+^.

**Fig. 4:**
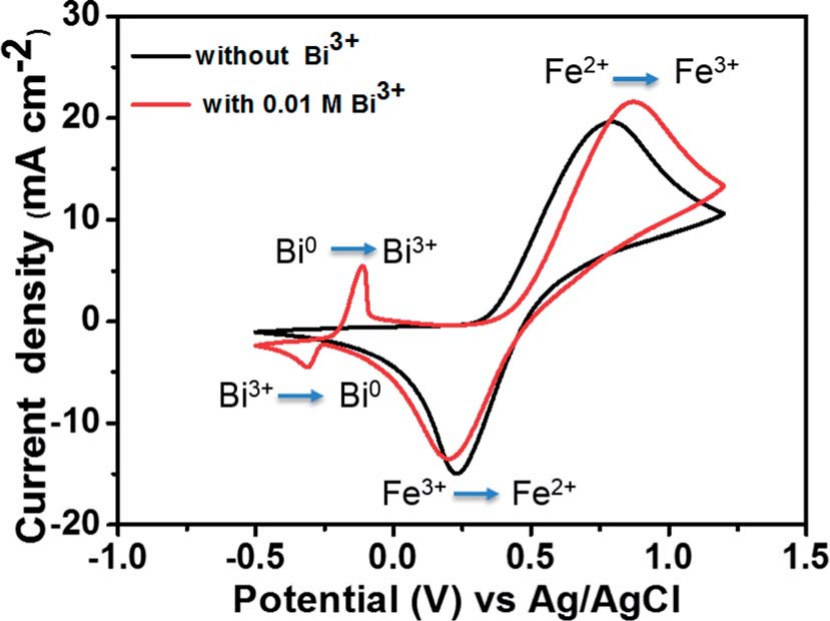
CV curves of 1.5 M FeCl_2_ in a 3 M HCl using GC electrode with and without 0.01 M Bi^3+^ at a scan rate of 50 mV s^−1^.

To compare the electrochemical activities, a CV test was performed with and without the addition of Bi_2_O_3_. Fig. 4 presents the CV curves of the Fe^2+^/Fe^3+^ redox couple on a GC working electrode with and without the addition of Bi^3+^ at a scan rate of 50 mV s^−1^. The matching reactions of oxidation–reduction couples are marked in the figure. The standard potential of Bi^3+^/Bi^0^ (46 mV *vs.* Ag/AgCl) is detected in the CV curve along with that of the Fe^2+^/Fe^3+^ redox couple owing to the addition of Bi^3+^. The addition of Bi^3+^ ions has no influence on the oxidation–reduction reaction of Fe^2+^/Fe^3+^. This is because the Bi metal will be oxidized to Bi^3+^ ions prior to the oxidation of Fe^2+^ to Fe^3+^, indicating that Bi in the form of ions has no catalytic effect toward Fe^2+^/Fe^3+^ redox couple.^25,26^

**Fig. 5:**
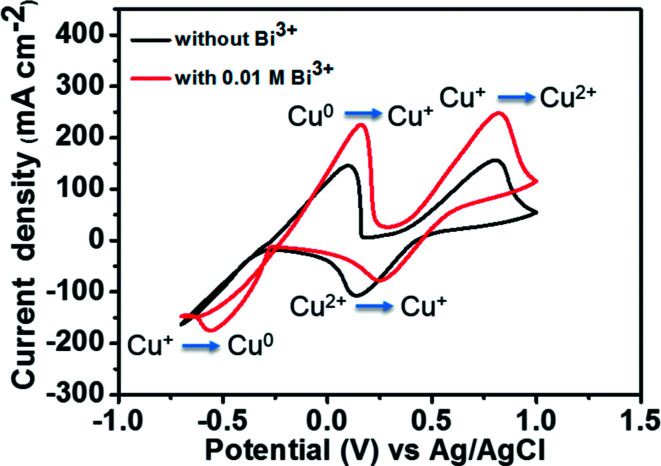
CV curves of 1.8 M CuCl in a mixed supporting electrolyte (2.4 M CaCl_2_ + 2.4 M HCl) solution using GC electrode with and without 0.01 M Bi^3+^ at a scan rate of 50 mV s^−1^.

Fig. 5 depicts the CV curves of 1.8 M CuCl in 2.4 M HCl/2.4 M CaCl_2_ electrolyte on a GC working electrode with and without adding Bi_2_O_3_ at a scan rate of 50 mV s^−1^. The corresponding redox pair reactions are marked in the figure. For the electrolyte without Bi^3+^, the peak potential separation corresponding to the Cu^+^/Cu^2+^ redox couple is 0.66 V. However, after the addition of 0.01 M Bi^3+^ to the electrolyte, the redox peak potential separation declines to 0.57 V. Moreover, in the case of Cu^0^/Cu^+^ redox couple, both oxidation and reduction peaks are clearly observed after adding Bi^3+^ as compared to the pristine electrolyte. This result indicates that after adding Bi^3+^, the copper redox couples have better electrochemical activity and reversibility. The reason is that the bismuth metal will be electrodeposited onto the negative GC electrode prior to the reduction of Cu^2+^ to Cu^+^, suggesting that the Bi metal in place of Bi^3+^ ions has a catalytic effect towards copper redox reactions.^27,28^ To investigate the catalytic effect of the Bi ions towards copper redox reactions, we measured the diffusion coefficients and rate constants for Cu^0^/Cu^+^ couple with and without adding Bi_2_O_3_. The results are listed in Table 1. The standard rate constant of Cu^0^/Cu^+^ with addition of Bi_2_O_3_ (7.33 × 10^−4^ cm s^−1^) is greater than that without adding Bi_2_O_3_ (4.87 × 10^−4^ cm s^−1^), indicating that Cu^0^/Cu^+^ with Bi_2_O_3_ has smaller overpotentials under the same conditions.^19^

**Table 1:** Comparison of the diffusion coefficients and rate constants for Cu^0^/Cu^+^ couple with and without adding Bi_2_O_3_

Parameters	Without Bi^3+^	With 0.01 M Bi^3+^
Diffusion coefficient (*D*_O_)	7.40 × 10^−8^ cm^2^ s^−1^	1.74 × 10^−7^ cm^2^ s^−1^
Rate constant (*k*^0^)	4.87 × 10^−4^ cm s^−1^	7.33 × 10^−4^ cm s^−1^

**Fig. 6:**
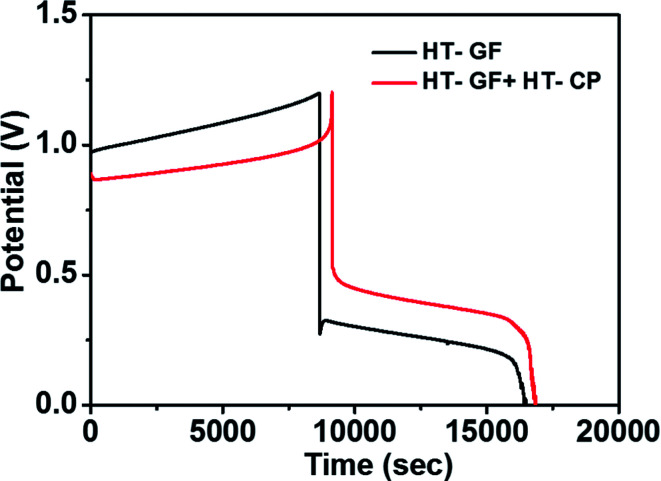
Charge–discharge curves of Fe/Cu RFB cells with HT-GF and HT-GF + HT-CP at a current density of 20 mA cm^−2^.

A charge–discharge cycling performance test was conducted using a Fe/Cu RFB single cell with Nafion 117 as a membrane to demonstrate the effect of the carbon electrode on the electrochemical performance of the Fe/Cu RFB cell. Fig. 6 presents the charge–discharge profiles for the cells with microwave heat treated GF (HT-GF) and microwave heat treated GF + microwave heat treated one layer CP (HT-GF + HT-CP) electrodes at a constant current density of 20 mA cm^−2^. The VRFB cell with the HT-GF + HT-CP electrode exhibits a longer charge–discharge time, lower charge voltage plateau, and higher discharge voltage plateau than the HT-GF, which results in higher energy and voltage efficiency. This is because more oxygen-containing functional groups are introduced onto the carbon fiber surface during the microwave pretreatment process,^29,30^ which can facilitate the Fe^2+^/Fe^3+^and Cu^+^/Cu^0^ redox reactions. Moreover, in the case of the HT-GF + HT-CP electrode, the subsequent electrodeposition of Cu metal onto the GF surface is lower, as shown in the ESI (Fig. S1b†), leading to higher discharge and lower charge voltage.^6,31^ The CE, EE, and VE data obtained from Fig. 6 are listed in Table 2.

**Table 2:** Summary of the efficiencies of two cells obtained at 20 mA cm^−2^

Cells with	CE (%)	EE (%)	VE (%)
HT-GF	88.77	21.05	23.71
HT-GF + HT-CP	84.10	34.43	40.94

**Fig. 7:**
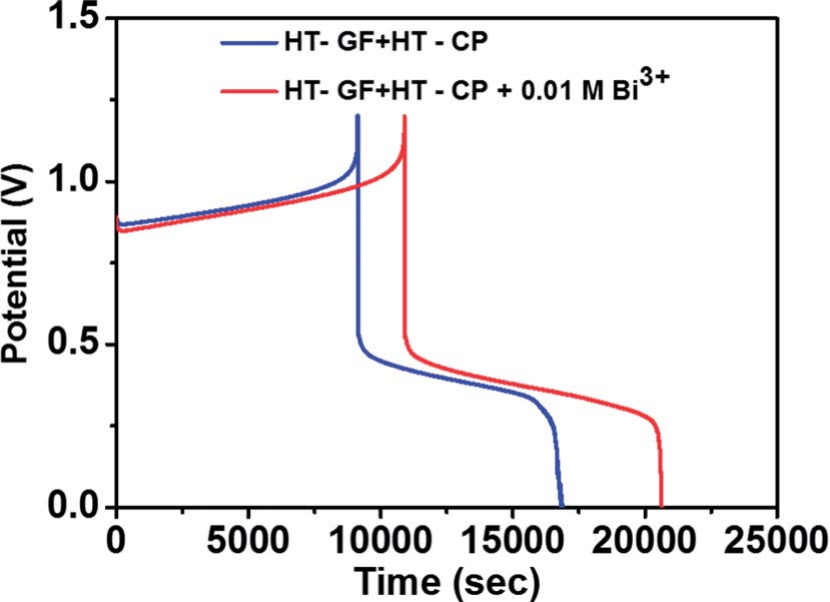
Charge–discharge curves of a Fe/Cu RFB with and without 0.01 M Bi^3+^ in the electrolytes at a current density of 20 mA cm^−2^.

To further investigate the effect of adding Bi^3+^ in the electrolytes on the performance of the Fe/Cu RFB single cell, charge/discharge cycling curves were recorded with and without 0.01 M Bi^3+^. Fig. 7 shows the charge–discharge curves of a Fe/Cu RFB in the absence and presence of Bi^3+^ in the electrolytes at a current density of 20 mA cm^−2^. The CE, EE, and VE data obtained from the curves shown in Fig. 7 are listed in Table 3. It is found that the cell containing Bi^3+^ in electrolytes reveals higher CE and EE than the cell without Bi^3+^. This is because after adding Bi^3+^, no Cu metal precipitation was observed on the electrode surface (see Fig. S1c†), which indicated that the electrodeposition process was potentially reversible on the electrode material.

**Table 3:** Summary of the efficiencies of two cells obtained at 20 mA cm^−2^

Cells with	CE (%)	EE (%)	VE (%)
HT-GF + HT-CP	84.10	34.43	40.94
HT-GF + HT-CP + 0.01 M Bi^3+^	89.18	35.24	39.52

**Fig. 8:**
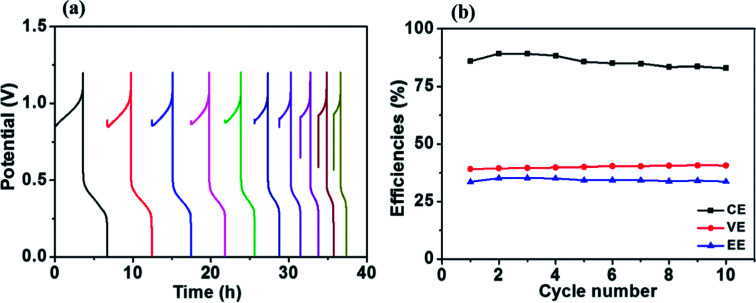
(a) Charge–discharge cycling curves and (b) stability test of the Fe/Cu RFB with 0.01 M Bi^3+^ at a current density of 20 mA cm^−2^.

Fig. 8a presents charge–discharge cycling curves of a Fe/Cu RFB with 0.01 M Bi^3+^ in the electrolytes at 20 mA cm^−2^. The resultant efficiencies obtained from Fig. 8a as a function of cycle number are plotted in Fig. 8b. As can be observed in Fig. 8b, the efficiencies of the battery are stable over 10 cycles, which demonstrates that Fe/Cu RFB has good stability on the HT-GF + HT-CP electrode after adding Bi^3+^ in the electrolytes.

In brief, we propose a novel, abundant, low-cost, and environmentally benign Fe/Cu RFB, which employs Fe^2+^/Fe^3+^ and Cu^+^/Cu^0^ as the positive and negative electrolytes, respectively. We investigated the effect of GF electrode modification and the addition of Bi^3+^ in the electrolytes on the charge–discharge performance of the Fe/Cu RFB. The results confirmed that the cell containing Bi^3+^ in electrolytes revealed higher CE (89.18%) and EE (35.24%) than the cell without Bi^3+^ (CE = 84.10% and EE = 34.43%) at a current density of 20 mA cm^−2^. This is because the presence of Bi^3+^ avoids precipitation of Cu metal on the electrode surface, which indicates that the electrodeposition process is potentially reversible on the electrode material, thus leading to enhanced performance of the battery.

Cost is probably the most important and fundamental issue of energy storage systems for a broad market penetration. The key costs of RFBs are the active material stored in the electrolyte and the electrochemical cell.^32^ Therefore, we considered only the electrolyte and stack material costs, while the cost of the remaining balance-of-plant was not considered. In the case of Fe/Cu RFB, we estimate that the cost of the electrolyte is around $33 (kW h)^–1^ of energy storage, which is much lower than that in the VRFB system. Considering the price of the materials used in the stack, we estimate that the stack has a price of around $100–150 m^−2^, which corresponds to a system cost of $170–230 (kW h)^−1^. The VRFB, which is the most developed aqueous RFB, is estimated to have a vanadium material cost of $189 (kW h)^−1^ and a system cost of $325 (kW h)^−1^.^6^ By considering these values, in combination with the lower electrolyte cost we place the Fe/Cu RFB system well ahead of the VRFB in terms of attractiveness for practical applications.

As we already estimated, the system cost of Fe/Cu RFB is about $170–230 (kW h)^−1^, while Li-ion batteries, for example, have a system cost about $400–600 (kW h)^−1^,^1^ which is clearly still too high for broad market penetration. The high cost is primarily attributed to the high cost of materials (lithium). Hence, Li-ion batteries are more expensive than Fe/Cu RFB. In general, for stationary applications, the Li-ion batteries of current interest for vehicle applications may face a few challenges. First and foremost, the cost of the Li-ion batteries that are targeted for vehicle applications is 2–5 times higher on a kW h basis than that for the stationary applications.^33^ In addition, the targeted performance parameters of the Li-ion batteries may not be good enough for stationary applications. For example, stationary applications generally require a longer cycle and calendar life than that for vehicle applications. A further challenge of Li-ion batteries for stationary applications is in safety and reliability. As for transportation applications, the Li-ion batteries must be safe and reliable. This becomes particularly important for grid applications for scale-up to MW levels.

## 4. Conclusions

In summary, a novel, abundant, low-cost, and environmentally benign Fe/Cu RFB on the basis of Fe^2+^/Fe^3+^ redox couple as the positive electrolyte and Cu^+^/Cu^0^ redox couple as the negative electrolyte in a flow cell was successfully developed for large-scale energy storage applications. The effect of GF electrode modification and addition of Bi^3+^ in the electrolytes on the charge–discharge performance of the Fe/Cu RFB were investigated. The results confirm the following. (1) The VRFB cell with the HT-GF + HT-CP electrode exhibited longer charge–discharge time, lower charge voltage plateau, and higher discharge voltage plateau than the HT-GF, which results in higher energy and voltage efficiency. This is because more oxygen-containing functional groups were introduced on the carbon fibers surface during the microwave pretreatment process, which facilitated the Fe^2+^/Fe^3+^ and Cu^+^/Cu^0^ redox reactions. (2) The cell containing Bi^3+^ in electrolytes reveals higher CE (89.18%) and EE (35.24%) than the cell without Bi^3+^ (CE = 84.10% and EE = 34.43%) at a current density of 20 mA cm^−2^. This is because the presence of Bi^3+^ avoids precipitation of Cu metal on the electrode surface, which indicates that the electrodeposition process is potentially reversible on the electrode material, thus leading to enhanced performance of the battery. Furthermore, the efficiencies of the battery are stable over 10 cycles, which demonstrates that Fe/Cu RFB has good stability on the HT-GF + HT-CP electrode after adding Bi^3+^ in the electrolytes. However, the energy efficiency of our proposed battery is still low. Further investigation is still needed to improve the energy efficiency by optimizing parameters such as the electrolytes' composition, cell component materials, and operating temperatures.

## Conflicts of interest

There are no conflicts to declare.

## Supplementary Material

RA-008-C7RA12926B-s001
