# Supplementary material for: The effect of adding Bi3+ on the performance of a newly developed iron–copper redox flow battery
Source: RSC Adv. 2018 Feb 23;8(16):8537–43. doi: 10.1039/c7ra12926b (PMC9078536; doi:10.1039/c7ra12926b)
Supplement: RA-008-C7RA12926B-s001 [file RA-008-C7RA12926B-s001.pdf]

Electronic Supplementary Material (ESI) for RSC Advances.
This journal is © The Royal Society of Chemistry 2018

Supporting Information

The effect of adding Bi^{3+} on the performance of a newly developed iron-copper redox flow battery

Daniel Manaye Kabtamu,[1] Guan-Yi Lin,[1] Yu-Chung Chang,[1] Huseh-Yu Chen,[1] Hsin-Chih Huang,[1] Ning-Yih Hsu,[2] Yi-Sin Chou,[2] Hwa-Jou Wei,[2] Chen-Hao Wang*[1]

[1]*Department of Materials Science and Engineering, National Taiwan University of Science and Technology, 10607, Taipei, Taiwan.*

[2]*Institute of Nuclear Energy Research, Atomic Energy Council, Executive Yuan, 32546, Taoyuan, Taiwan.*

**Corresponding author, E-mail: chwang@mail.ntust.edu.tw*

Tel: +886-2-2730-3715; Fax: +886-2-2737-6544

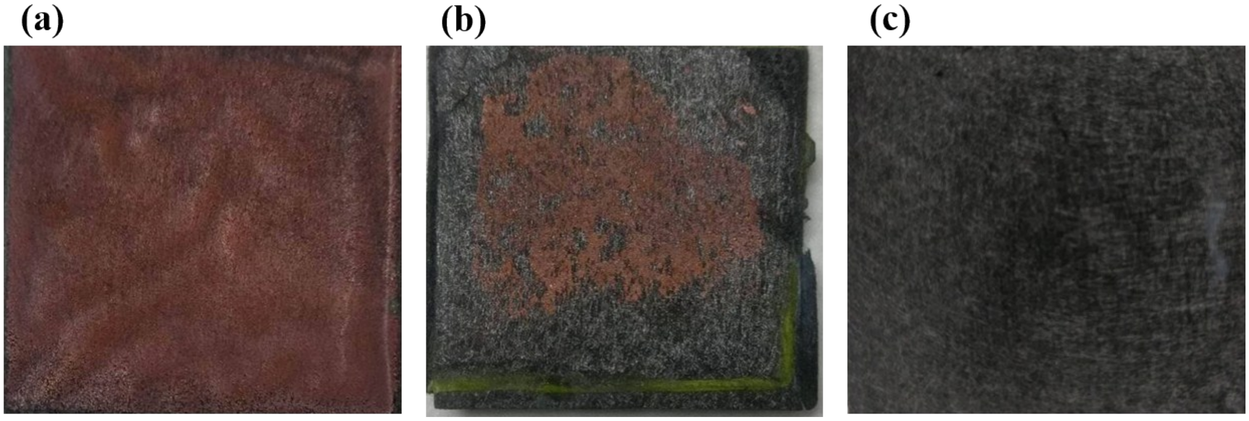

Fig. S1. Photographs of (a) HT-GF, (b) HT-GF+ HT-CP without Bi^{3+}, and (c) HT-GF + HT-CP with 0.01 M Bi^{3+} after charge-discharge cycling measurements.

To make sure the reaction which is proceed in the negative electrode (cathode side), we studied the property of CuCl, which is difficult to dissolve in water.When the Cu^{+} ion is added into water, it forms CuCl(s) precipitation. Based on this property of CuCl, we set 5 stages for the charge-discharge test, which shows in **Fig. S2**. At each stage, we took 5 ml of anolyte and then added 20 ml DI water to observe the change of precipitation. The results from each stage refer to **Fig. S3 and S4**. From stage 1 to stage 2, there is a little decrease of CuCl precipitation (white), which is due to the reduction of Cu^{+} to Cu(s). From stage 2 to stage 3, no precipitation is observed because of the Cu^{+} ion is totally transformed into Cu metal. From stage 3 to stage 4, there is some CuCl participation occurred owing to Cu metal started to become Cu^{+}. From stage 4 to stage 5, more precipitation is observed because of larger amount of Cu(s) becomes Cu^{+}. From the above explanation, we conclude that the redox reaction is takes place between Cu(s) and $Cu^{+.}$

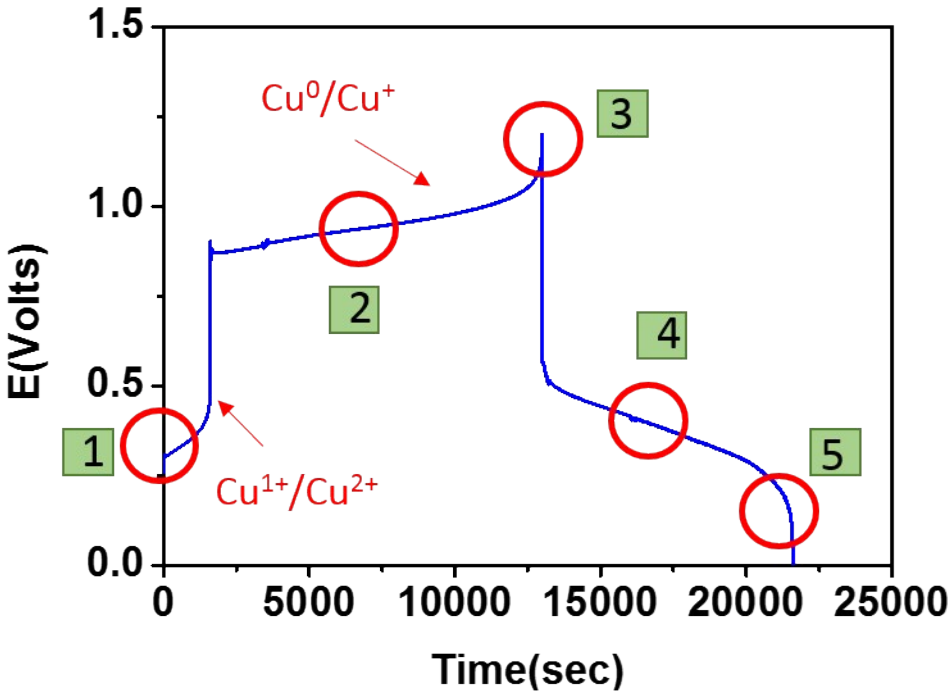

Fig. S2..The 5 stages of the charge-discharge curve.

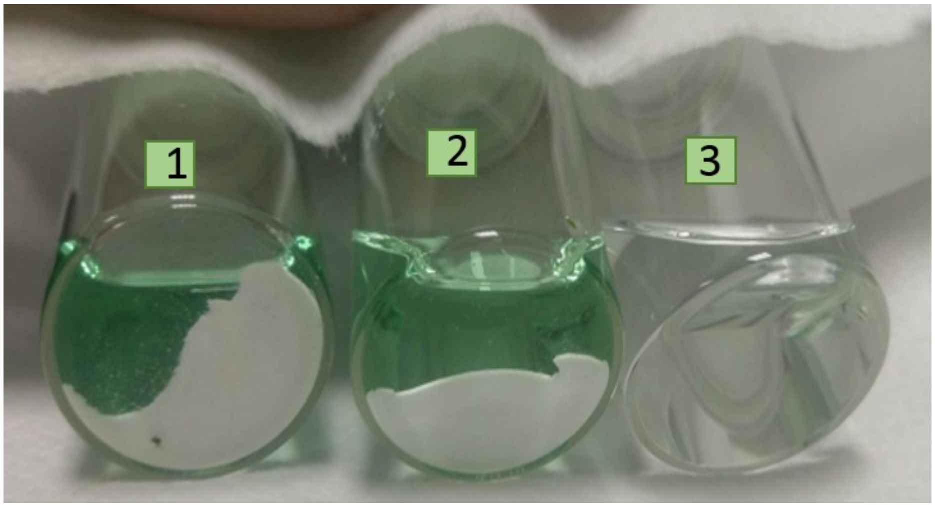

Fig. S3. The result of the stage 1, 2 & 3 of the charge-discharge curve.

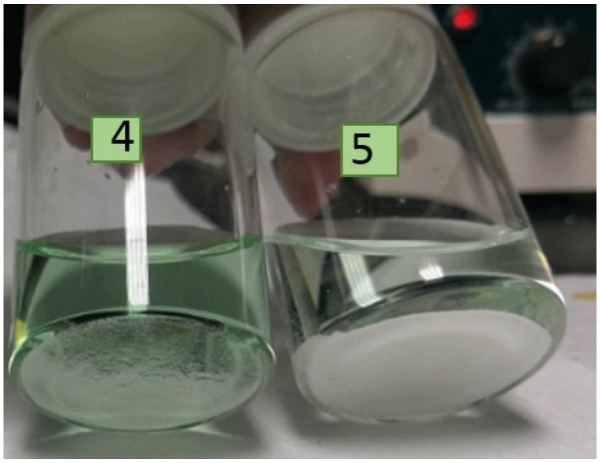

Fig. S4. The result of the stage 4 &5 of the charge-discharge curve.
